# TANK-Binding Kinase 1 in the Pathogenesis and Treatment of Inflammation-Related Diseases

**DOI:** 10.3390/ijms26051941

**Published:** 2025-02-24

**Authors:** Lu Hui, Xiaolin Chen, Mengke Huang, Yongmei Jiang, Ting Liu

**Affiliations:** 1 Department of Laboratory Medicine, West China Second University Hospital, Sichuan University, No. 20, Section 3, Renmin Road South, Chengdu 610041, China; huiluscu@163.com (L.H.); xiaolin_chen090180@163.com (X.C.); huangmengke77@163.com (M.H.); 2 Key Laboratory of Obstetric & Gynecologic and Pediatric Diseases and Birth Defects of Ministry of Education, Sichuan University, Chengdu 610041, China; 3 State Key Laboratory of Biotherapy and Cancer Center/National Collaborative Innovation Center for Biotherapy, Sichuan University, Chengdu 610041, China

**Keywords:** TBK1, inflammation, immunity, metabolism, cancer

## Abstract

TANK-binding kinase 1 (TBK1) is a key signaling kinase involved in innate immune and inflammatory responses. TBK1 drives immune cells to participate in the inflammatory response by activating the NF-κB and interferon regulatory factor signaling pathways in immune cells, promoting the expression of pro-inflammatory genes, and regulating immune cell function. Thus, it plays a crucial role in initiating a signaling cascade that establishes an inflammatory environment. In inflammation-related diseases, TBK1 acts as a bridge linking inflammation to immunity, metabolism, or tumorigenesis, playing an important role in the pathogenesis of immune-mediated inflammatory diseases, metabolic, inflammatory syndromes, and inflammation-associated cancers by regulating the activation of inflammatory pathways and the production of inflammatory cytokines in cells. In this review, we focused on the mechanisms of TBK1 in immune cells and inflammatory-related diseases, providing new insights for further studies targeting TBK1 as a potential treatment for inflammation-related diseases. Thus, optimizing and investigating highly selective cell-specific TBK1 inhibitors is important for their application in these diseases.

## 1. Introduction

The kappa B kinase inhibitor (IKK) family comprises multifunctional serine/threonine protein kinases that are major regulators of inflammation and innate immunity [1]. The IKK family includes IKKα, IKKβ, IKKγ, IKKε, and TANK-binding kinase 1 (TBK1), which share 25–65% sequence homology and structural similarity [2,3,4]. Based on structural variability, the IKK family can be divided into classical and non-classical IKK kinases. The classical IKK kinases include IKKα and IKKβ, which interact with IKKγ to form the classical IKK complex. This complex regulates various biological processes, such as apoptosis, gene expression, growth, and metabolism, and is believed to connect chronic inflammation with cancer progression [1,5]. Non-classical IKK kinases include IKKε and TBK1, also known as IKK-associated kinases (IKKs), because their structural domains are similar to those of classical IKK kinases. TBK1 shares up to 65% sequence homology with IKKε, resulting in significant functional overlap between the two proteins. They form a heterodimer and are involved in the interferon regulatory factor (IRF)3/7 and NF-κB signaling pathways, which regulate innate immunity, inflammatory responses, and tumor formation [6,7]. Notably, high expression of IKKε is restricted to specific cells, such as hepatic stellate cells, adipocytes, and adipose tissue macrophages [8]. TBK1 is constitutively expressed in most cell types, including immune cells, histiocytes, and other cells, and is significantly elevated in disease states [9,10]. For example, in systemic lupus erythematosus (SLE), TBK1 expression in patient immune cells increases with disease activity, particularly in CD4^+^ T cells and myeloid cells [11]. In hepatocellular carcinoma (HCC), TBK1 expression levels are also significantly higher than in normal liver tissue and are associated with a poorer prognosis [12,13]. Moreover, TBK1, which is widely expressed in cells, exerts broad-spectrum functions in the organism. In immune cells, TBK1 is mainly involved in immune response, antiviral response, and inflammatory response [11,14]. While in various non-immune cells such as liver, kidney, and tumor cells, TBK1 may be more involved in tumorigenesis, cell proliferation, and growth [12,13,15]. This garners attention to TBK1 as an emerging research target.

In addition to its widespread expression in cells, TBK1 is remarkable for its involvement in the regulation of many signaling pathways. It receives excitation signals from pattern recognition receptors (PRRs), phosphorylates, and activates downstream signaling pathways. The NF-κB signaling pathway is a major regulator of inflammation and the earliest identified signaling pathway involved in TBK1. Studies have revealed that TBK1 binds directly to the stimulatory region in TANK, activating the NF-κB signaling pathway mediated by tumor necrosis factor (TNF) receptor-associated factors 2, NF-κB-inducing kinase, and IKK complexes [16,17]. Furthermore, more than 99% homology has been identified between mouse and human TBK1 proteins, demonstrating that TBK1 was highly conserved in mammals; thus, research on TBK1 in animal disease models provides a valuable reference to human diseases [16]. The IRF3 signaling pathway is another well-known pathway in which TBK1 plays a prominent role. Furthermore, TBK1 plays an important role in antiviral immunity and inflammatory regulation. It induces the phosphorylation of IRF3, leading to IRF3 dimerization and nuclear translocation, following which it binds to the interferon (IFN)-stimulated response element in the nucleus, leading to Toll-like receptor (TLR)-mediated type I IFN responses [18,19]. Interestingly, accumulating data suggest that IRF3 and NF-κB signaling pathways are able to act synergistically. The innate immune system induces the production of type I IFN and pro-inflammatory cytokines (TNF-α) through the TBK1-IRF3/NF-κB signaling pathways, which play important roles in innate immunity and inflammation [20]. With the deep study of TBK1, it has also been found that TBK1 participates in IRF7, AKT, MAPK, mTOR, RIPK, and other signaling pathways, which regulate immune response, inflammation, metabolism, autophagy, apoptosis, cell proliferation, mitosis, and insulin signaling, thus playing a wide range of roles in the pathogenesis of autoimmune, inflammatory and metabolic diseases, and cancer [21,22,23,24,25,26,27].

Thus, TBK1, as a multifunctional protein kinase, has great potential as a drug target in the future. Among the various biological functions of TBK1, our focus is on its role in regulating inflammation. Increasing evidence suggests that inflammation is the foundation of numerous pathological processes, which is associated with almost all human diseases [28,29]. Inflammatory cells and mediators are involved in a wide range of biological processes during disease, such as autoimmunity, tissue remodeling, oxidative stress, cell proliferation, vascular regeneration, metabolism, aging, and neurological functions [29,30,31,32,33,34]. However, although many excellent reviews mention inflammation when discussing the various roles of TBK1 in diseases, such as innate immunity, cancer immunity, and autophagy, specific reviews discussing its role in regulating inflammation are lacking [27,35,36,37]. Therefore, a better understanding of the mechanisms by which TBK1 regulates inflammation in immune cells and diseases is important for investigating therapeutic strategies in diseases characterized by inflammation-based features (inflammation-associated diseases).

## 2. Role of TBK1 in Immune Cells

**Figure 1:**
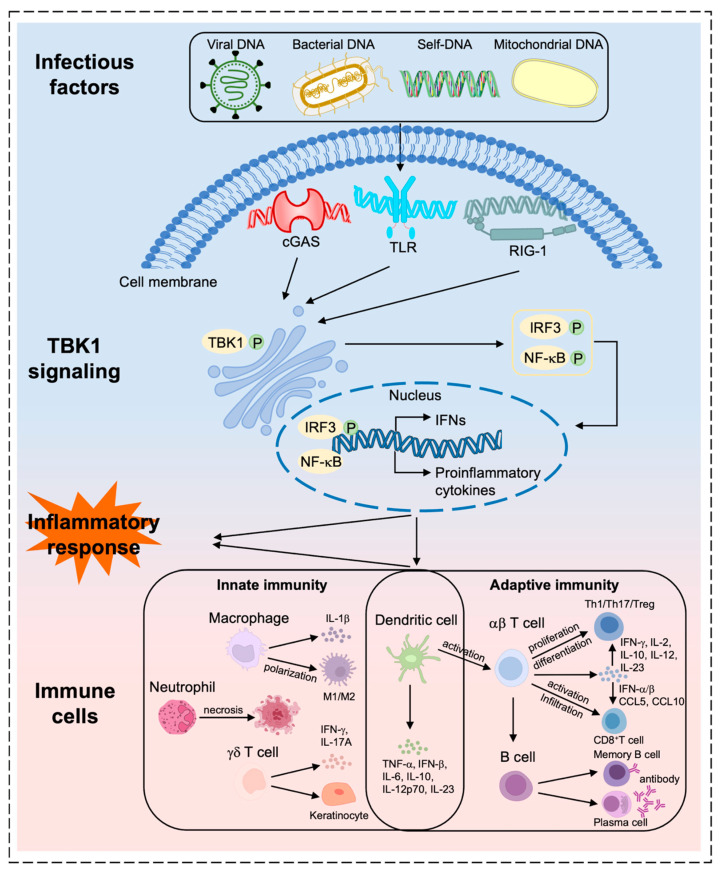
**Regulation of different immune cells by TBK1.** Stimulated by infectious factors, PRR excitation signals (cGAS, TLR, and RIG-1) activate the TBK1-NF-kB/IRF3 signaling pathway, which regulates the function of different immune cells and together participate in the inflammatory response.

Immune cells are key regulators of inflammation and are involved in the onset and abatement of inflammatory responses [38,39]. TBK1 signaling is essential for IFNα/β and inflammatory cytokine expression in immune cells [40]. TBK1 receives signals from PRRs expressed by innate immune cells to activate NF-κB and IRF transcription factors and initiate adaptive immune signaling, which promotes the expression of pro-inflammatory genes and drives innate immune cells to participate in the inflammatory response [41,42,43]. Also, TBK1 regulates immune cell functions, including macrophage polarization, dendritic cell (DC) maturation, B-cell differentiation, and the activation, proliferation, and differentiation of inflammatory T cells [44,45,46]. These findings suggest that TBK1’s function in immune cells is pivotal in initiating signaling pathways that drive the establishment of an inflammatory environment (Figure 1). Therefore, we first explored the regulation of various immune cells by TBKI to gain deeper insights into the mechanisms through which it regulates the inflammatory response in disease.

### 2.1. Macrophages

Macrophages are one of the major cells regulating the inflammatory response during the innate immune response that mediate the production of inflammatory mediators in disease [47]. The critical role of TBK1 in macrophage-mediated inflammatory response has been demonstrated. TBK1 is involved in the TLR-IRF3 signaling pathway in macrophages, and its downstream IFN-α/β signaling is critical for host defense against bacterial (group B Streptococcus, Pneumococcus, and Escherichia coli) infections [48]. In addition to the IRF signaling pathway, TBK1 attenuates the induction of pro-inflammatory cytokines (especially interleukin [IL]-1β) by acting in macrophages to inhibit the NF-κB and MAPK signaling pathways [49]. Furthermore, evidence suggests that TBK1 regulates the inflammatory response of tissue-specific macrophages. In the central nervous system (CNS), activation of the STING-TBK1-IRF3 signaling pathway inhibits the activation of microglia to a pro-inflammatory phenotype and the secretion of pro-inflammatory cytokines [50]. In the liver, TBK1 deficiency in Kupffer cells inhibits endotoxin tolerance and promotes the production of pro-inflammatory cytokines production [51]. In the intestine, TBK1 negatively regulates the expression of IL-1β expression in macrophages in intestinal epithelial cells (IEC) [52]. In the skeletal system, TBK1 mediates the genesis and differentiation of osteoclasts by regulating the NF-κB, MAPK, and AKT signaling pathways [21,53]. In addition, TBK1 is involved in macrophage polarization. Studies have confirmed that myeloid-specific TBK1 deficiency promotes M1 macrophage polarization in the adipose and liver tissues of non-alcoholic fatty liver disease (NAFLD) mice, promoting adipose tissue inflammation and hepatitis [49]. Thus, TBK1 regulates various tissue-specific macrophage-mediated inflammatory responses and polarization through the IRF/NF-κB/MAPK signaling pathway.

### 2.2. Neutrophils

Neutrophils, which are polymorphonuclear granulocytes (PMNs), are the first cells recruited to sites of infection and inflammation [54]. Neutrophil-intrinsic TBK1 activates the downstream IRFs/NF-κB signaling pathway in response to PRR stimulation, which promotes the expression of inflammatory cytokines (IFNγ and IL-12) and ROS production, participating in inflammatory responses in the lungs [55]. Emerging evidence shows that knockdown- or inhibitor-induced TBK1 dysfunction increases PMN necrotic apoptosis, resulting in increased lung inflammation [56]. The overproduction of hydrolases and reactive oxygen species by PMNs is one of the causes of local peripheral tissue damage [57]. Furthermore, PMN necrotic apoptosis can exacerbate the inflammatory response by releasing damage-associated molecular patterns [58]. Therefore, TBK1 regulates neutrophil function, such as chemotaxis and ROS generation, and targeting TBK1 can reduce PMN-associated inflammation.

### 2.3. Dendritic Cells

Dendritic cells (DCs) are important mediators that connect innate and adaptive immunity, initiating protective pro-inflammatory and tolerogenic immune responses [59]. Thus, TBK1 is essential for DC development and function and a key factor in DC involvement in inflammatory responses [27,60,61]. With regard to innate immunity, after Mycobacterium bovis infection, the cGAS/STING/TBK1/IRF3 signaling pathway is activated in bone-marrow-derived dendritic cells (BMDCs), promoting the maturation of the cells and significantly increasing in the expression of co-stimulatory molecules (CD40, CD80, CD86 and MHC II) and inflammatory cytokines (TNF-α, IL-6, IL-10, and IL-12p70) [62]. This effect has been validated using various TBK1 inhibitors, including BX795, CYT387, and amlexanox (ALX) [44,61,63]. While exploring herpes simplex virus vaccines, researchers found that BX795 prevented the maturation of DCs stimulated by viral infection, significantly decreasing the expression of CD40 and CD86. It also blocked IRF3 phosphorylation and reduced the expression of inflammatory cytokines, such as IFN-β, IL-1β, IL-6, and TNF-α, in DCs [63]. Furthermore, CYT387 inhibits the gene expression of pro-inflammatory cytokines (TNF, IL-1β, IL-6, and IL-12p70) in mature DCs by interfering with the AKT and NF-κB signaling cascade, which has shown therapeutic effects in sepsis mouse models [61]. Consistent with this, in a mouse model of experimental autoimmune encephalomyelitis (EAE), ALX treatment reduced the phosphorylation of IRF3 and AKT in BMDCs and splenic DCs, inhibited the gene expression of CD80 and CD86 in splenic DCs, and decreased IL-12 and IL-23 secretion [44]. With regard to adaptive immunity, TBK1 in DCs regulates autoimmunity and antitumor immunity by modulating the abnormal production of inflammatory T cells through antigen presentation [44,64]. These findings indicate that upon activation by PRRs in DCs, TBK1 regulates inflammatory responses in innate and adaptive immunity via downstream signaling.

### 2.4. T Cells

Gamma-delta (γδ) T cells are a specific type of innate immune T cells that play a key role in regulating the inflammatory response and antitumor immunity at the mucosal barrier [65]. Activation of the STING-TBK1 pathway promotes the secretion of IFN-γ and IL-17A by γδ T cells, which are also involved in pro-inflammatory crosstalk with keratinocytes, thereby modulating oral immune responses in oral lichen planus [66].

Unlike γδ T cells, αβ T cells are the predominant T cell subpopulation involved in the adaptive immune response, usually expressing CD4 or CD8 lineage markers [65]. CD4^+^ T cells further differentiate into various helper T-cell (Th) subpopulations in response to different cytokine stimuli and continue to participate in the inflammatory response and produce cytokines. It has been demonstrated that TBK1 initiates adaptive immune signaling and participates in inflammation by regulating CD4^+^ T-cell activation, proliferation, differentiation, and inflammatory cytokine production in cellular immunity. In the intestine, the IEC expression of TBK1 inhibits MT1 expression, which prevents colonic inflammation and tumorigenesis by preventing the expression of IL-1β in macrophages in the intestinal lamina propria; consequently, the differentiation of lamina propria naïve CD4^+^ T cells into Th17 cells is inhibited [52]. In contrast, in a mouse model of EAE, ALX reduced the absolute number of mouse splenic CD4^+^ T cells and inhibited the secretion of IL-2, IFNγ, IL-12, and IL-23, thereby suppressing the BMDC-mediated activation and proliferation of Th1 and Th17 cells. Furthermore, ALX treatment increased the secretion of regulatory T cells (Treg) and IL-10 in the CNS and spleen. The combined effect of inflammatory T cells and DCs reduced inflammatory infiltration and demyelination in spinal cord tissues, ultimately reducing the severity of EAE in mice [44]. Also, in the tumor microenvironment (TME), activation of the TBK1 downstream signaling pathway can contribute to inflammation by enhancing the infiltration and cytotoxic function of CD8^+^ T cells [67]. The activation of the TBK1/IRF3 signaling pathway in the BRCA1-deficient triple-negative breast cancer and small cell lung cancer models increased IFN-α/β and pro-inflammatory chemokine (CCL5, CXCL10) production. This would enhance CD8^+^ T-cell infiltration and activation via antigen presentation, which promotes immune responses and participates in the establishment of an inflammatory TME [45,68]. Evidence from these studies supports the involvement of TBK1 in regulating the immune activation of different types of T cells in innate and cellular immunity, which then participates in inflammation in different ways, such as cell activation, proliferation, cytokine production, cell infiltration, and cytotoxicity.

### 2.5. B Cells

The role of TBK1 in humoral immunity has also been confirmed in several studies. TBK1-driven inducible T-cell co-stimulator (ICOS) signaling is required to generate germinal center (GC)-derived memory B and plasma cells and thymus-dependent antibody responses. Inhibition of TBK1 reduces humoral autoimmunity through the ICOS-driven GC pathway [40]. Moreover, elevated p-TBK1 in B cells during GC differentiation regulates IRF4/BCL6 expression by limiting CD40 and B-cell receptor activation through NF-κB/AKT signaling pathways. After the knockdown of TBK1 in B cells, the generated memory B cells fail to achieve aseptic immunity upon re-infection, suggesting that TBK1 determines the fate of B cells in promoting durable humoral immunity [46]. In addition, TBK1 negatively regulates immunoglobulin (Ig)A class switching by attenuating non-classical NF-κB signaling; moreover, B-cell-specific TBK1 deficiency leads to aberrant antigen-induced IgA, elevated steady-state concentrations of serum IgA, and nephropathy-like symptoms in aged mice [69]. In conclusion, TBK1 is involved in inflammatory signaling pathways in humoral immunity to regulate germinal center B cells and IgA class switching.

## 3. Role of TBK1 in Inflammation-Related Diseases

**Figure 2:**
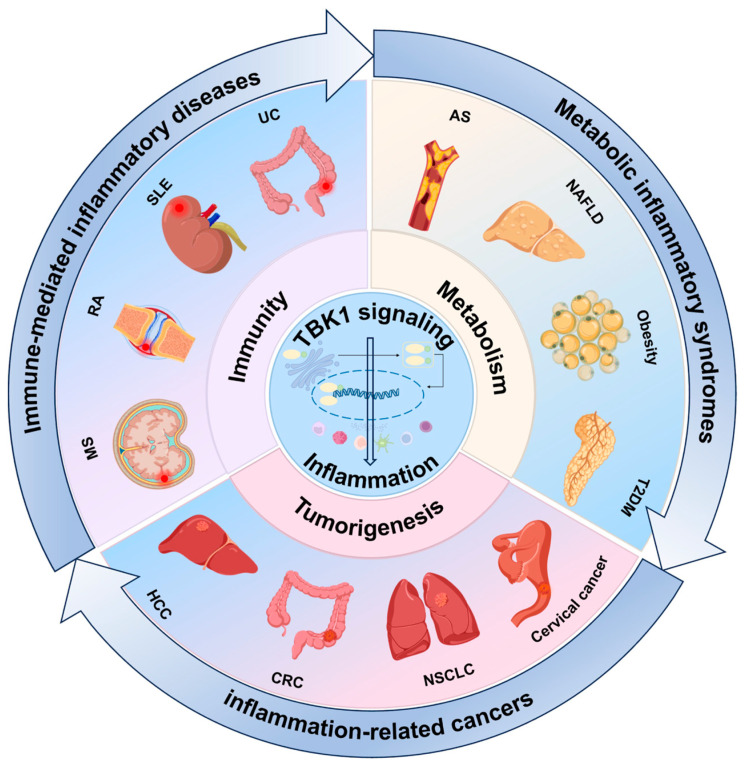
**Role of TBK1 in key mechanisms of inflammation-associated diseases.** Inflammation is a central link in the pathogenesis and clinical manifestations of a variety of inflammation-associated diseases. TBK1 plays a key role in a variety of inflammation-associated diseases by linking inflammation to immunity, metabolism, or oncogenesis through the activation of various signaling pathways and the regulation of immune cell function.

Inflammation runs through the core of the pathogenesis and clinical manifestations in many inflammation-related diseases. TBK1 acts as a bridge connecting inflammation to immunity, metabolism, or tumorigenesis in these diseases (Figure 2). In the following paragraphs, we have classified inflammation-related diseases into three categories and discussed the mechanisms of TBK1 in immune-mediated inflammatory diseases (IMIDs), metabolic inflammatory syndromes (MIS), and inflammation-associated cancers.

### 3.1. Immune-Mediated Inflammatory Diseases

IMIDs result from an immune-mediated inflammatory cascade and affect multiple tissues and organs, displaying both inflammatory and autoimmune traits [70,71]. Accumulating studies have shown that early and effective control of inflammation levels and modulation of immune imbalance can effectively improve the prognosis of patients with IMIDs [71,72,73]. TBK1 is a key signaling kinase involved in the regulation of inflammation and innate immunity, closely associated with the pathogenesis of IMIDs, such as rheumatoid arthritis (RA), autoinflammatory arthritis, ulcerative colitis (UC), systemic lupus erythematosus (SLE), asthma, and multiple sclerosis (MS) [11,74,75,76,77,78,79].

In RA, TBK1 regulates synovial inflammation by activating the IFN signaling pathway in rheumatoid fibroblast-like synoviocytes, inducing the production of pro-inflammatory factors, and regulating the activation of inflammatory T-cells and macrophage recruitment to the synovium [74,75]. In mouse experiments in autoinflammatory arthritis, it was found that interfering with TBK1 recruitment to STING-induced circulating monocytes prevented the activation of the NF-κB and IRF3 signaling pathways and eliminated the production of pro-inflammatory cytokines [78]. Consistent with this, in UC patients, one study detected significantly elevated TBK1 mRNA levels in the inflamed intestinal mucosa compared to those in normal mucosa. Blocking the activation of the TLR-TBK1-IRF3/7 signaling pathway in the intestinal epithelium attenuated colonic inflammation in a mouse model of DSS-induced colitis [76]. In contrast to TBK1 inhibition, myeloid TBK1 knockout exacerbated colonic inflammation and tissue damage in mice, indicating its vital role in preventing colonic inflammation [49]. In SLE patients, elevated TBK1 mRNA levels have been detected in B cells, T cells, and myeloid cells. Additionally, targeting the TBK1-IRF3 signaling pathway in macrophages reduced SLE-induced inflammation and mortality in mouse experiments and bone marrow-derived macrophages (BMDM) [11,77]. TBK1 was involved in the pathogenesis of asthma induced by house dust mite-induced endoplasmic reticulum stress in one study. P-TBK1 promoted the phosphorylation of NF-κB/STAT6 in vivo and in vitro, thereby enhancing airway mucus formation [80]. The TBK1/IRF3 signaling pathway has also been a key factor in IL-33 release from lung fibroblasts in cyclic guanosine monophosphate–adenosine monophosphate-induced allergic asthma [81]. By blocking the TBK1 pathway with ALX or si-TBK1, asthmatic mice exhibited a reduction in airway cuprocytes and attenuated allergic inflammation in the lungs [80,81]. These studies suggest that TBK1 regulates inflammatory responses in IMIDs by activating inflammation-associated signaling pathways, promoting cytokine production and immune cell activation.

In a mouse model of EAE that was developed to investigate the pathogenesis of MS, the absence of TBK1 promoted T-cell activation and hindered T-cell migration [79]. Consequently, the migration of effector T cells from the draining lymph nodes to the CNS was blocked, resulting in the attenuation of neuroinflammation. This confirms that TBK1 also mediates the crosstalk between immune response and inflammation in IMIDs by mediating immune cell activation and subsequent migration to peripheral organs. Furthermore, a study by Dan Li et al. reported a therapeutic role in animal disease models of IMIDs, including RA, SLE, psoriasis, and atopic dermatitis, using CS12192, a novel JAK3/JAK1/TBK1 inhibitor [82]. The mechanism of TBK1 in several other IMIDs may be of further interest. Therefore, it is reasonable to conclude that TBK1 is a potential therapeutic target in IMIDs.

### 3.2. Metabolic Inflammatory Syndromes

MIS is defined as a group of metabolic disorders in which metabolic inflammation induced by metabolites (e.g., free fatty acids and lipopolysaccharides) leads to tissue and organ damage. MIS includes obesity, type 2 diabetes mellitus (T2DM), NAFLD, and atherosclerosis (AS), which are often coexisting or concurrent [83,84]. Insulin resistance, metabolic inflammation, and metabolic abnormalities are the common pathological characteristics of the MIS [85,86,87,88]. MIS can be ameliorated after administering anti-inflammatory therapy [89]. Emerging evidence suggests that TBK1 is a key node in regulating the potential connection between metabolism and inflammatory response in metabolic diseases [25,49,90,91].

In obesity and T2DM, TBK1 mainly plays a key role in regulating metabolic inflammation associated with glucose metabolism [26,92]. Significant increases in adipose tissue TBK1 have been detected in obese mouse models [25,93,94]. The activation of the TBK1-mTORC/IL-17 axis in the adipose tissues of diet-induced obese mice reduces the expression of anti-inflammatory genes in these tissues, attenuating insulin resistance and improving glycemic control in these mice [93,95]. Consistent with this finding, several studies have attempted cell-specific knockout of TBK1 in obese mice, including myeloid and adipocyte. The results consistently showed that TBK1KO activates the MAPK/NF-κB signaling pathways, which induces the secretion of pro-inflammatory cytokines, resulting in adipose tissue inflammation and insulin resistance enhanced in mice [49,90]. However, the results of pharmacological interventions differed from the knockout results. Inhibition of TBK1 with ALX in HFD mice reduced IL-17-induced adipose tissue inflammation and improved diet-induced obesity, fatty liver, glucose, and lipid metabolism in mice [25,95]. A clinical study in obese and diabetic patients has also shown that ALX treatment improved glucose metabolism, insulin sensitivity, and hepatic steatosis in patients. The effectiveness of the drug was associated with higher serum C-reactive protein levels and adipose tissue inflammation [96]. In addition, systematic loss-of-function studies on TBK1 indicate that the protective effect of ALX on metabolism in obese mice stems from the inhibition of TBK1 [97].

In NAFLD, TBK1 is involved in both glucose metabolism and lipid metabolism-related inflammatory regulation [91]. In glucose metabolism, inhibition of the TBK1-mediated NF-κB-MCP1 signaling pathway reduces pro-inflammatory macrophage recruitment and improves insulin resistance, resulting in efficacy in NAFLD mice [98]. In lipid metabolism, inhibition of the hepatic macrophage STING-TBK1-NF-κB pathway improved HFD-induced hepatic lipid deposition by attenuating macrophage inflammation and reducing the release of inflammatory cytokines [99]. Similarly, reduced activation of TBK1-AMPK signaling in hepatocytes is reported to prevent hepatic inflammatory injury and lipid accumulation [100]. In HFD-fed hepatocyte-specific TBK1 knockout mice, increased hepatic steatosis exacerbation and increased expression of inflammation-related genes were detected, along with reduced fatty acid oxidation, exacerbating hepatic lipid accumulation [91,101].

In AS, TBK1 prevents AS by modulating lipid metabolism and inflammatory responses [102,103]. The pharmacological inhibition of ALX significantly improved dyslipidemia and reduced inflammatory gene expression in the livers of HFD mice. Simultaneous down-regulation of inflammatory genes, especially TGF-β signaling in smooth muscle cells, may lead to attenuated vascular smooth muscle cell proliferation and migration. This can inhibit the monocyte–macrophage invasion of the vascular endothelium, leading to phagocytosis of cholesterol and the formation of foamy cells that alleviate the dysfunction of aortic vascular cells [102]. Another drug, tetrandrine, reduced macrophage inflammation and attenuated AS in HFD mice by inhibiting the STING-TBK1-NF-κB inflammatory signaling pathway [103]. Furthermore, in clinical studies of diabetes-induced AS, hyperglycemia activates TBK1, thereby exacerbating AS by inducing IL17/IL10-mediated inflammatory responses via TBK1-HIF-1α signaling in macrophages and dendritic cells [104].

These studies consistently demonstrated that TBK1 is activated in high-glucose/high-lipid environments in MIS, inducing the activation of multiple signaling pathways to regulate immune cell-mediated inflammatory response, glucose metabolism, and lipid metabolism. Overall, the dual regulation of inflammatory responses and metabolism by TBK1 has significant potential for the study of new therapeutic agents. However, since TBK1 affects metabolic reprogramming in different cell types, further investigations are necessary to determine the regulatory mechanism of TBK1 on MIS [94].

### 3.3. Inflammation-Associated Cancers

Studies have shown that approximately 20% of malignant tumors worldwide are associated with pre-existing chronic inflammation, as evidenced in both clinical studies and animal experiments [105,106,107,108]. Contrary to acute inflammation that activates antitumor immunity, chronic inflammation promotes tumorigenesis and progression mainly by inducing the recruitment of immune-suppressive cells, including myeloid-derived suppressor cells, tumor-associated macrophages (TAM), Treg cells, regulatory B cells (Breg), regulatory NK cells, and tolerogenic dendritic cells, thereby disrupting inflammatory signaling pathways and creating a TME [109]. Typical inflammation-associated cancers include hepatocellular carcinoma (HCC), cholangiocarcinoma (CCA), gastric cancer, lung cancer, colon cancer, cervical cancer, and bladder cancer [107,108,110,111,112]. In recent years, potential targets for cancer treatment using anti-inflammatory therapies have been extensively investigated and shown therapeutic efficacy [113,114,115]. Among them, TBK1 has been shown to be a drug target of interest in inflammation-associated cancers [35,36,116,117].

TBK1 mediates immunosuppression by promoting tumor inflammation, thereby promoting tumor growth. In HCC, TBK1 expression was significantly higher in cancer tissues than in normal tissues and correlated with the expression levels of hepatic inflammatory markers (liver fibrosis and platelet/albumin ratio) [12,13]. Further studies revealed that TBK1 promoted the establishment of inflammatory TME by activating the NF-κB signaling pathway, increasing the release of inflammatory cytokines (type I interferon, IL-6, and IL-17) from HCC cells, recruiting TAMs, and inhibiting the activation of tumor-infiltrating CD8+ T cells [12,13]. Meanwhile, TBK1 expression was positively correlated with immunosuppressive cell markers, such as CD37, ITGAM, FUT39, CCL33, CD4, IL-2, FOXP68, CCR10, and STAT3B, inducing HCC immunosuppression and promoting HCC progression by maintaining an inflammatory phenotype [13]. Reasonably, the treatment of a primary HCC model established using GSK 8612 (a highly selective TBK1 inhibitor) in mice with chronic liver inflammation significantly attenuated HCC progression in C57 BL/6 mice [13]. Similar evidence in support of this idea has been seen in intestinal tumor models, wherein intestinal epithelial cell-specific TBK1 knockdown increased the expression of the pro-inflammatory immune regulator, metallothionein 1 (MT1), leading to the expression of IL1β in macrophages and differentiation of pro-inflammatory Th17 cells, resulting in inflammation and colorectal tumorigenesis [52]. Additionally, TBK1 promoted glycolysis through chronic inflammation-induced mTORC 1, thus supporting CRC development [118]. Studies on lung cancer have also shown that TBK1 signaling was essential for the survival of KRAS-driven lung cancer cells, which is dependent on TBK1-AKT/mTOR signaling-mediated tumor growth and immunosuppression [119,120,121]. Clinical studies have identified high expression levels of TBK1 in the tumor tissues of stage 1 non-small cell lung cancer (NSCLC) patients that were significantly correlated with poor prognosis [122]. Further studies based on mouse lung cancer models and human cancer cell lines demonstrated that TBK1 signaling-regulated CCL 5 and IL-6 promote KRAS-driven NSCLC by affecting lung cancer cell proliferation and the local inflammatory microenvironment [123,124,125]. In addition, TBK 1 promotes cervical cancer progression by interacting with Golgi transporter protein 1B, which enhances NF-κB signaling-mediated tumor inflammation [126,127]. These studies confirmed the association of TBK1 with inflammation-associated cancers and clarified the promotional role of TBK1 in chronic inflammation-mediated tumor development.

TBK1 has a regulatory role in the growth and proliferation of cancer cells in inflammation-associated cancers. In rat and mouse CCA, TBK1 levels were increased with the development of spontaneous carcinogenesis, promoting the epithelial-mesenchymal transition by binding to β-catenin. Inhibition of TBK1 expression by specific siRNA or drugs inhibits the growth, migration, and invasion of human hepatobiliary carcinoma cells [128]. In bladder cancer cells and tissues, the upregulation of TBK1 expression was observed. Knockdown or BX795 inhibition of TBK1 effectively controlled the proliferation and migration of bladder cancer cells by attenuating AKT phosphorylation [129]. In NSCLC, TBK1 is a key factor in the KRAS-induced oncogenic transformation of mouse embryonic fibroblasts; the activation of TBK1 by upstream signaling inhibits the apoptosis of cancer cells [130]. Consistent with this, TBK1 promotes anti-apoptotic signaling by activating the NF-κB signaling pathway, which also plays a crucial role in the survival of KRAS-mutant lung cancer cells [119]. In addition, increased TBK1 expression has been detected in gastric cancer tissues [131]. However, the abovementioned studies did not explore the effect of TBK1 on tumor inflammation. Thus, further studies may be important for early intervention in the progression from chronic inflammation to cancer.

In conclusion, targeted inhibition of TBK1 can effectively treat various cancers, thus indicating TBK1 as a potential target for cancer therapy [13,128,129,132]. Importantly, in inflammation-associated cancers, we discuss the effect of TBK1 on tumors from the perspective of its regulation of tumor inflammation, which also provides new ideas for the treatment of such diseases.

## 4. Therapeutic Potential of Targeting TBK1

The current therapeutic strategies target TBK1 at two levels: (1) Since total knockout of TBK1 caused embryonic lethality in mice, cell-specific knockout of TBK1 was performed, including adipocytes, hepatocytes, IEC, dendritic cells, B cells, T cells, and myeloid cells. (2) Many inhibitors targeting TBK1 have been applied in various animal models and clinical trials of inflammation-related diseases (Table 1).

### 4.1. Cell-Specific Knockout of TBK1

Accumulating studies have shown that the specific TBK1 knockout in various cell types (mainly categorized into histiocytes and immune cells) is involved in the regulation of inflammatory responses. TBK1-specific knockout promotes tissue inflammation in inflammation-related diseases. In adipocytes, specific knockout of TBK1 attenuates the inhibitory effect of AMPK on the NF-κB signaling pathway, resulting in NF-κB overactivation, which promotes macrophage inflammation in adipose tissue [90]. In hepatocytes, TBK1-specific knockout promoted the increased expression of inflammation-related genes in the liver of HFD-fed mice under fasting [91,101]. In IECs, TBK1-specific knockout increased the expression of the pro-inflammatory immunomodulator MT1, which hyperactivated NF-κB in mice, leading to the expression of IL1β in macrophages and differentiation of pro-inflammatory Th17 cells, thereby promoting inflammation in the colonic tissues of mice [52].

Studies in various immune cells also support the idea that TBK1-specific knockout modulates inflammation. In mouse splenic DCs, TBK1-specific knockout resulted in the increased expression of co-stimulatory molecules (CD80 and CD86), which promote the immunostimulation of T cells, leading to the earlier onset and increased severity of EAE [64]. In B cells, TBK1-specific knockout rendered memory B cells unable to achieve aseptic immunity upon re-infection, impairing humoral immunity and resulting in the development of human IgA nephropathy-like disease in mice [46]. In myeloid cells, TBK1-specific knockout exacerbated colonic inflammation and tissue damage in mice by activating the MAPK/NF-κB signaling pathways and inducing the secretion of pro-inflammatory cytokines in macrophages and neutrophils [49]. However, in the Influenza A virus infection model, TBK1-specific knockout in myeloid cells resulted in reduced gene expression levels of IRF3 and NF-κB in mouse lung tissues, decreased alveolar recruitment of inflammatory macrophages, reduced production of inflammatory cytokines, and attenuated inflammatory response to viral infection [127]. In T cells, TBK1-specific knockout promoted T cell activation and differentiation, producing higher levels of Th1 and Th17 cells. However, in another trial, TBK1-specific knockout in T cells impaired the migratory function of T cells, leading to lymph node T-cell retention and a decrease in the number of T cells in the CNS, which inhibited the occurrence of EAE in mice [79]. These studies suggest that TBK1 regulates the inflammatory response differently in different cell types, especially immune cells. Thus, it might be necessary to consider the cell types that play a major role in inflammatory-related diseases and the functioning mechanisms of immune cells.

### 4.2. TBK1 Inhibitors

Several TBK1 inhibitors have been evaluated. Among these inhibitors, some have been shown to correlate with inflammatory responses in animal models of inflammation-related diseases or clinical trials, such as ALX, WEHI-112, Compound II, and DMXD-011. ALX, a small molecule inhibitor acting on TBK1 and IKKε, is the most representative of these drugs and has been evaluated in the clinic. In a 25-week trial in patients with type 2 diabetes, oral ALX significantly reduced serum HbA1c and fructosamine levels, and the effectiveness of ALX was primarily related to the severity of adipose inflammation [96]. ALX has also shown therapeutic properties against adipose inflammation in animal models of NAFLD, obesity, and type 2 diabetes. In an obese mouse model, ALX significantly reduced the exudation of inflammatory macrophages in white adipose tissue, inhibited the expression of key inflammatory genes TNF-α, CCL12, CCL13, and Emr1 in adipose tissue, and promoted the expression of the anti-inflammatory cytokine IL-10 in serum [25]. In addition, ALX also significantly down-regulated the mRNA levels of inflammatory cytokines TNF-α, IL-1β, and IL-6 as well as chemokines CCL2, CXCL1, CXCL2 in the injured liver by inhibiting TBK1/IKKε. This suppressed the neutrophil infiltration and NF-κB phosphorylation in the liver, thereby alleviating acetaminophen-induced hepatic fibrosis and acute liver injury in mice [133]. WEHI-112 is a semi-selective TBK1 inhibitor that acts on both IKKε and JAK. In mouse experiments, WEHI-112 was shown to eliminate the TBK1-dependent type I IFN response and inhibit the expression of IFN-β and IL-6. Meanwhile, WEHI-112 treatment selectively abrogated the clinical and histological features of antibody-dependent collagen-induced arthritis (CIA) [134].

Compound II is a 6-aminopyrazolopyrimidine derivative and a TBK1-specific inhibitor that specifically targets the TBK1- IRF3 pathway. In the study by Hasan et al., TBK1 expression was significantly higher in immune cells from SLE patients than those in healthy controls [11]. By inhibiting TBK1 with Compound II, the activation of the TBK1/IRF3 signaling pathway in BMDM and peritoneal macrophages was attenuated, and the expression of inflammatory cytokines in peritoneal cells was reduced, improving mouse survival. DMXD-011 is a pyrimidine-based compound with high potency and selectivity for TBK1. It is involved in inhibiting inflammatory cytokine production and non-toxicity in animal models of CIA. Efforts are underway to further improve this compound as a treatment for SLE and interferon-related diseases [24].

These TBK1 inhibitors have shown promise in the treatment of inflammation-related diseases, and the specificity of TBK1 as an inflammatory coordinator is an important reason why it is a potential target for immunotherapy and disease intervention. However, their application has some limitations. First, due to the high structural similarity, most currently reported small molecule inhibitors are dual-targeted inhibitors of TBK1 and IKKε [135]. Second, cell-specific knockout studies suggest that the unselective inhibition of all cell types by inhibitors may have unpredictable results due to differences in the regulation of inflammatory responses by TBK1 in different cell types. Therefore, future research targeting TBK1 could be directed toward highly selective cell-specific TBK1 inhibitors. At the single-cell level, cell-specific inhibitors can target the expression of specific genes or proteins to achieve selective inhibition of specific cells in accordance with differences in the gene expression patterns of different cell types.

## 5. Conclusions

In this review, we focused on the mechanisms of TBK1 as a key signaling kinase in immune cells and inflammation-related diseases, providing new insights for further studies targeting TBK1 for the treatment of these diseases. TBK1 serves as a critical link between inflammation and its crosstalk with immunity, metabolism, and tumorigenesis. Additionally, it plays an important role in the pathogenesis of various inflammation-related diseases by regulating the activation of inflammatory pathways and the production of inflammatory cytokines. However, poor specificity and role-dependent cell type limit the broad use of TBK1 inhibitors. Therefore, optimizing and studying highly selective cell-specific TBK1 inhibitors are important for their application in inflammation-related diseases.

## Figures and Tables

**Table 1:** Targeting strategies for TBK1 in inflammation-related disease models.

Targeting Strategies		Disease Models	Key Findings
Cell-specific knockout of TBK1	Adipocytes	obesity	promoting macrophage inflammation in adipose tissue [90]
	Hepatocytes	NAFLD	promoted the increased expression of inflammation-related genes in the liver of HFD-fed mice [91,101]
	IECs	Colon cancer	promoting differentiation of pro-inflammatory Th17 cells and colonic inflammation [52]
	DCs	EAE	promoting the immune stimulation of T cells by DCs [64]
	B cells	Malaria infection model	impairing humoral immunity [46]
	Myeloid cells	Experimental Colitis, Influenza A virus infection model	inducing the secretion of pro-inflammatory cytokines in macrophages and neutrophils, exacerbating colonic inflammation [49] decreasing alveolar recruitment of inflammatory macrophages, reducing production of inflammatory cytokines, and attenuating inflammatory response to viral infection [127]
	T cells	EAE	promoting T cell activation and differentiation, impairing the migratory function of T cells [79]
TBK1 inhibitors	Amlexanox	NAFLD, obesity, and type 2 diabetes	the effectiveness in patients with type 2 diabetes is related to the severity of adipose inflammation [96] reducing the exudation of inflammatory macrophages in white adipose tissue [25] suppressing the neutrophil infiltration in the liver [133]
	WEHI-112	CIA	selectively abrogating the clinical and histological features of antibody-dependent CIA [134]
	Compound II	SLE	reducing immune activation of BMDMs and peritoneal macrophages and decreasing the expression of inflammatory cytokines in peritoneal cells [11]
	DMXD-011	CIA	inhibiting inflammatory cytokine production [24]

## Abbreviations

TBK1: tank-binding kinase 1; IKK: inhibitor of kappa B kinase; NEMO: NF-κB essential modifier; IKKs: IKK-associated kinases; IRF: interferon regulatory factor; PRRs: pattern recognition receptors; TNF: tumor necrosis factor; TLRs: Toll-like receptors; IFN: interferon; DC: dendritic cell; IL: interleukin; CNS: central nervous system; IEC: intestinal epithelial cells; PMNs: polymorphonuclear granulocytes; ALX: amlexanox; AMPK: AMP-activated protein kinase; γδ T cells: Gamma-delta T cells; Th cell: helper T cell; Ig: immunoglobulin; ICOS: inducible T cell co-stimulator; GC: germinal center; IMIDs: mediated inflammatory diseases; MIS: metabolic inflammatory syndromes; RA: rheumatoid arthritis; UC: ulcerative colitis; SLE: systemic lupus erythematosus; MS: multiple sclerosis; BMDM: bone marrow-derived macrophages; EAE: experimental autoimmune encephalomyelitis; AS: atherosclerosis; T2DM: type 2 diabetes mellitus; NAFLD: non-alcoholic fatty liver disease; HFD: high-fat diet; TAM: tumor-associated macrophages, Treg cells: regulatory T cells, Breg cells: regulatory B cells; TME: tumor microenvironment; HCC: hepatocellular carcinoma, CCA: cholangiocarcinoma; MT: metallothionein; NSCLC: non-small cell lung cancer; CIA: collagen-induced arthritis.
